# Prevalence of intentions to receive monkeypox vaccine. A systematic review and meta-analysis

**DOI:** 10.1186/s12889-023-17473-y

**Published:** 2024-01-02

**Authors:** Darwin A. León-Figueroa, Joshuan J. Barboza, Mario J. Valladares-Garrido, Ranjit Sah, Alfonso J. Rodriguez-Morales

**Affiliations:** 1 Facultad de Medicina Humana, Universidad de San Martín de Porres, Chiclayo, 15011 Peru; 2 Unidad de Revisiones Sistemáticas y Meta-análisis, Universidad San Ignacio de Loyola, Lima, 15046 Peru; 3 Universidad Continental, Lima, 15046 Peru; 4 Oficina de Epidemiología, Hospital Regional Lambayeque, Chiclayo, 14012 Peru; 5 Department of Microbiology, Institute of Medicine, Tribhuvan University Teaching Hospital, Kathmandu, 44600 Nepal; 6 Department of Microbiology, Hospital and Research Centre, Dr. D. Y. Patil Medical College, Dr. D. Y. Patil Vidyapeeth, Pune, 411018 Maharashtra India; 7 Department of Public Health Dentistry, Dr. D.Y. Patil Dental College and Hospital, Dr. D.Y. Patil Vidyapeeth, Pune, 411018 Maharashtra India; 8 Master of Clinical Epidemiology and Biostatistics, Universidad Cientifica del Sur, Lima, 15067 Peru; 9 Gilbert and Rose-Marie Chagoury School of Medicine, Lebanese American University, Beirut, 1102 Lebanon

**Keywords:** Monkeypox, Vaccine, Vaccine hesitancy, Vaccine intentions, Mpox

## Abstract

**Background:**

Immunization, as a preventive strategy against infectious diseases, has consolidated its position as a fundamental pillar in the field of public health. Therefore, the present study aimed to determine the prevalence of the intention to receive the monkeypox (Mpox) vaccine.

**Methods:**

A systematic review and meta-analysis of the available evidence was performed using five databases (PubMed, Scopus, Web of Science, Embase, and ScienceDirect) with a search strategy until July 24, 2023. Data analysis was performed in R software version 4.2.3. The quality of the included cross-sectional studies was assessed using the “JBI-MAStARI”. In addition, a subgroup analysis by population and continent was developed.

**Results:**

Twenty-nine cross-sectional articles with a total sample of 52 658 participants were included. The pooled prevalence of intention to vaccinate against Mpox was 61% (95% CI: 53–69%; 52,658 participants; 29 studies; I^2^ = 100%). In the subgroup analysis, the intention to be vaccinated against Mpox according to continents was 64% (95% CI: 53–74%; 13,883 participants; 17 studies; I^2^ = 99%) in Asian countries, 43% (95% CI: 39–47%; 1538 participants; 3 studies; I^2^ = 53%) in African countries, 62% (95% CI: 45–78%; 35,811 participants; 6 studies; I^2^ = 99%) in European countries, and 63% (95% CI: 32–89%; 1426 participants; 3 studies; I^2^ = 99%) in American countries. In the subgroup analysis on the intention to be vaccinated against Mpox, according to study subjects, it was 54% (95% CI: 45–62%; 10,296 participants; 11 studies; I^2^ = 99%) in the general population, 57% (95% CI: 33–79%; 3333 participants; 10 studies; I^2^ = 99%) in health care workers, and 76% (95% CI: 70–82%; 39,029 participants; 8 studies; I^2^ = 98%) in the lesbian, gay, bisexual, transgender, and intersex (LGBTI) community. In addition, as a secondary outcome, a prevalence of refusal of Mpox vaccination was found to be 22% (95% CI: 16–30%; 45,577 participants; 21 studies; I^2^ = 99%).

**Conclusion:**

The study highlights the importance of recognizing regional and subgroup disparities in Mpox vaccine willingness and refusal. It emphasizes the importance of employing strategies to achieve widespread vaccination coverage and safeguard public health worldwide.

**Terms used:**

Joanna Briggs Institute Meta-Analysis of Statistics Assessment and Review Instrument (JBI-MAStARI), Prospective International Registry of Systematic Reviews (PROSPERO), and Preferred Reporting Items for Systematic Reviews and Meta-Analyses (PRISMA).

**Supplementary Information:**

The online version contains supplementary material available at 10.1186/s12889-023-17473-y.

## Introduction

Within the current public health scenario, the prevention and control of emerging infectious diseases have acquired a fundamental role in the contemporary scientific and medical agenda [1, 2]. In response to these challenges, various strategies have been devised to address them; however, immunization has proven to be an invaluable tool to attenuate the spread of pathogens and safeguard the health of communities [3, 4]. In this context, the focus of the present research is directed towards an infectious agent of growing interest: the monkeypox virus [5].

Monkeypox (Mpox), caused by the monkeypox virus, is a viral disease belonging to the family Poxviridae [6]. Although once considered a rare disease of limited scope, the rapid spread of cases in a number of nations, both endemic and non-endemic, has triggered a global public health emergency [7]. The ability of the Mpox virus to induce death in humans ranges from 1 to 10%, highlighting the importance of assessing the population’s intention to vaccinate against this pathogen [5, 8].

The prevention of infectious diseases through immunization has been consolidated as a fundamental pillar of public health, having achieved the successful eradication of smallpox and a drastic decrease in the incidence of numerous vaccine-preventable diseases [9, 10]. However, to achieve optimal levels of community protection and prevent disease re-emergence, it is essential to understand the factors that influence vaccine acceptance [11, 12]. Intention to receive a vaccine is influenced by a complex interplay of sociodemographic, cultural, psychological, and risk perception variables [13, 14], highlighting the need for detailed research on population intention toward Mpox vaccination.

Therefore, the objective of the present investigation is to determine the prevalence of the intention to receive the Mpox vaccine. These findings could contribute to the development of more effective communication strategies and public health policies, guiding the prevention of Mpox and providing relevant information to strengthen preparedness and response to possible future outbreaks [13].

## Materials and methods

### Protocol and registration

The process of this research has been duly recorded in PROSPERO (**CRD42023 447,971**), ensuring transparency and thoroughness in the protocol. The systematic review and meta-analysis adhered to the **PRISMA checklist** guidelines during its conduct (Table S1).

### Eligibility criteria

#### Inclusion criteria

All cross-sectional studies addressing the prevalence of the intention to vaccinate against Mpox were included. No limitations were applied regarding language, time period, or geographic location. However, only those studies that were fully available, included sample size details, and presented relevant data on any aspect related to the intention of vaccination against Mpox were incorporated.

#### Exclusion criteria

The studies whose research topics did not align with the objectives of our investigation were excluded, as were those that employed a different design than a cross-sectional study. Likewise, incomplete articles were rejected, either due to insufficient data or a lack of information on the desired results. Finally, an attempt was made to establish contact with the corresponding author via email; however, unfortunately, it was not possible.

### Information sources and search strategy

Two researchers conducted thorough searches in various renowned databases, including PubMed, Scopus, Embase, Web of Science, and ScienceDirect. To optimize the search, they used key terms such as “monkeypox”, “Mpox”, “vaccine”, and “attitude”. The specific search strategies employed for each database are detailed in Table S2. The initial search was conducted on July 1, 2023, and was updated on July 24, 2023.

### Study selection

The authors used the Rayyan tool to store and manage the results obtained from the search strategy. After removing duplicate articles, a preliminary selection of the remaining ones was carried out by reading titles and abstracts, following pre-established criteria. Subsequently, a comprehensive review of the full reports was conducted to determine their compliance with the inclusion criteria. Any discrepancies were resolved through discussions and consultations with a researcher.

### Main and secondary results of the study

This study addresses two fundamental variables: the main one, focused on the intention to be vaccinated against Mpox, and the secondary one, related to the refusal to be vaccinated against this disease. Both were delineated from the following question: Do you plan to be vaccinated against Mpox?

#### Intention to vaccinate against Mpox

The definition of this primary variable was based on responses related to willingness or likelihood to be vaccinated against Mpox. Participants’ decisions regarding vaccination against this disease highlight the importance of immunization, either as a preventive measure or in response to vaccine availability.

#### Refusal of the Mpox vaccination

The definition of this secondary variable was based on responses indicating the likelihood of not being vaccinated or refusing the Mpox vaccine.

### Quality assessment

Two independent researchers conducted the evaluation of the quality of the included cross-sectional studies using the “JBI-MAStARI” method. In the event of any discrepancies in the assessments, a third investigator was involved to resolve them. The studies were classified based on their quality scores as high (≥ 7 points), moderate (4 to 6 points), or low (< 4 points) [15] (Table S3).

### Data collection process and data items

Two expert researchers collected the relevant data from the selected articles. Then, they extracted the following details and recorded them in an Excel spreadsheet: the name of the primary author, publication year, country, sample size, study population, gender (male and female), prevalence of intent to vaccinate against Mpox, number of cases of intent to vaccinate against Mpox, prevalence of refusal to vaccinate against Mpox, number of cases of refusal to vaccinate against Mpox, type of survey, and date of data collection. Finally, a third researcher verified the extracted data to ensure its accuracy and eliminate any incorrect information.

### Data analysis

Firstly, the selected articles were entered into a Microsoft Excel spreadsheet for further analysis using R, version 4.2.3. The results were presented using narrative tables and graphs. The estimation of the joint prevalence of Mpox vaccination intent was conducted using the random-effects model with inverse variance weighting. To assess heterogeneity among the studies, the Cochrane Q statistic was used, and its quantification was performed using the *I*^*2*^ index. Values of 25%, 50%, and 75% were considered indicators of low, moderate, and high heterogeneity, respectively. In order to examine publication bias, funnel-shaped graphs were employed, and Egger’s regression test was applied. The presence of potential publication bias was considered when the *p*-value was less than 0.05.

Additionally, subgroup analyses were conducted based on the study population and continent. The presentation of the pooled prevalence of Mpox vaccination intent was done using a forest plot format, which included 95% confidence intervals.

## Results

### Study selection

**Fig. 1:**
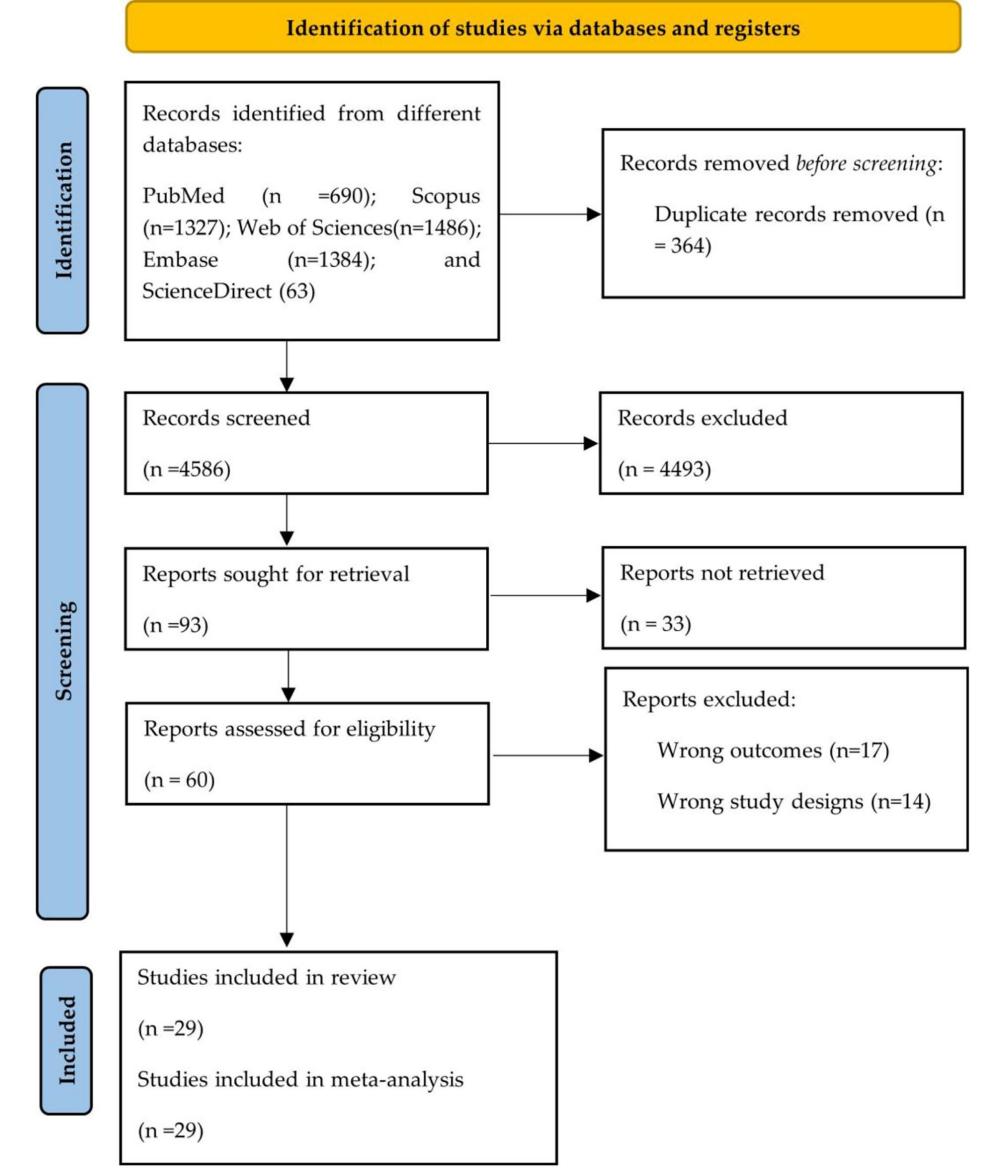
Study selection process based on the PRISMA flowchart

A total of 4950 articles were identified through systematic searches in five databases. After removing 364 duplicate records, 4586 articles were left for review. Subsequently, a thorough evaluation of the full texts (n = 60) was conducted, of which 29 studies fully met the eligibility criteria [16–44]. To visualize the study selection process, the detailed flow diagram in Fig. 1 is presented.

### Characteristics of the included studie

Table 1 summarizes the characteristics of the included studies [16–44]. This study encompassed 29 cross-sectional research articles, involving a total of 52,658 individuals from 19 countries, published between 2020 and 2023. Of the participant pool, 84.59% (n = 44,543) were men, while 15.26% (n = 8,036) were women. The questionnaires used for data collection were exclusively administered through online surveys, specifically tailored for diverse populations, including the general population, healthcare professionals, and the lesbian, gay, bisexual, transgender, and intersex (LGBTI) community [16–44].

**Table 1:** Characteristics of included studies on intention to vaccinate against monkeypox

Authors	Year	Study design	Country	Sample size (n)	Study population	Sex		Prevalence of vaccination intention	Participants with intention to be vaccinated	Survey Type	Refusal of monkeypox vaccination (n;%)	Data Collection Date
						**M**	**F**					
Araoz-Salinas JM, et al. (16)	2023	Cross sectional	Peru	373	LGBTI community	317*	23	88.5%	330	Online survey	43 (11.5%)	1 November 2022–17 January 2023
Mahameed H, et al. (17)	2023	Cross sectional	Jordan	495	Healthcare Workers	204	291	28.9%	143	Online survey	187 (37.8%)	January 2023
Wang B, et al. (18)	2023	Cross sectional	China	2135	General population	798	1337	68.8%	1468	Online survey	667 (31.2%)	30 August − 15 September 2022
Al-Mustapha AI, et al. (19)	2023	Cross sectional	Nigeria	822	General population	472	342	46.37%	339	Online survey	NR	16–29 August 2022
Fu L, et al. (20)	2023	Cross sectional	China	577	LGBTI community	577*	0	56.8%	328	Online survey	249 (43.2%)	10 August − 9 September 2022
Dukers-Muijrers NHTM, et al. (21)	2023	Cross sectional	Netherlands	1856	LGBTI community	1856*	0	81.5%	1512	Online survey	223 (12%)	22 July − 5 September 2022
Tran BX, et al. (22)	2023	Cross sectional	Vietnam	842	General population	239	595	65.4%	551	Online survey	13 (1.5%)	April - August 2022
Ghazy RM, et al. (23)	2023	Cross sectional	Ghana	605	General population	368	237	46.1%	279	Online survey	326 (53.9%)	27 November − 6 December 2022
Hong J, et al. (24)	2023	Cross sectional	China	1032	Healthcare Workers	266	766	90.12%	930	Online survey	102 (9.88%)	30 May – 1 August 2022
Jamaleddine Y, et al. (25)	2023	Cross sectional	Lebanon	493	General population	119	374	56.6%	279	Online survey	88 (17.9%)	6–20 September 2022
Dong C, et al. (26)	2023	Cross sectional	China	521	General population	264	257	76.40%	398	Online survey	35 (6.7%)	29 September 2022–5 October 2022
Chen Y, et al. (27)	2023	Cross sectional	China	154	LGBTI community	154*	0	63%	97	Online survey	57 (37%)	1–31 August 2022
Lounis M, et al. (28)	2023	Cross sectional	Algeria	111	Healthcare Workers	33	78	38.7%	43	Online survey	NR	28 June − 18 September 2022
Ahmed SK, et al. (29)	2023	Cross sectional	Iraq	510	General population	277	233	25.9%	132	Online survey	217 (42.5%)	27–30 July 2022
Riad A, et al. (30)	2022	Cross sectional	Czech Republic	341	Healthcare Workers	33	303	8.8%	30	Online survey	153 (44.9%)	September 2022
Zucman D, et al. (31)	2022	Cross sectional	France	155	LGBTI community	155*	0	66.4%	103	Online survey	52 (33.6%)	July - August 2022
Reyes-Urueña J, et al. (32)	2022	Cross sectional	Europe	32,902	LGBTI community	32,902*	0	82%	26,980	Online survey	2686 (8.2%)	30 July–12 August 2022
Bates BR, et al. (33)	2022	Cross sectional	United States	197	Physicians	113	69	48.3%	96	Online survey	101 (51.7%)	2–11 September 2022
Zheng M, et al. (34)	2022	Cross sectional	China	2618	LGBTI community	2618*	0	90.2%	2362	Online survey	NR	1–3 July 2022
Sahin TK, et al. (35)	2022	Cross sectional	Turkey	283	Physicians	117	166	31.4%	89	Online survey	85 (30%)	20 August–2 September 2022
Wang H, et al. (36)	2022	Cross sectional	Netherlands	394	LGBTI community	394*	0	70.01%	276	Online survey	NR	July 2022
Salim NA, et al. (37)	2022	Cross sectional	Indonesia	75	Healthcare workers	49	26	77.3%	58	Online survey	2 (2.70%)	2–5 August 2022
Riccò, M, et al. (38)	2022	Cross sectional	Italy	163	Physicians	57	106	64.4%	105	Online survey	NR	24–31 May 2022
Meo SA et al. (39)	2022	Cross sectional	Saudi Arabia	1020	General population	466	554	43.7%	446	Online survey	NR	15 May 2022–15 July 2022
Temsah MH, et al. (40)	2022	Cross sectional	Saudi Arabia	1546	General population	650	896	50.6%	782	Online survey	NR	27 May 2022–5 June 2022
Kumar N, et al. (41)	2022	Cross sectional	Pakistan	946	University students	432	514	67.7%	640	Online survey	148 (15.6%)	15–30 October 2022
Lin GSS, et al. (42)	2022	Cross sectional	Malaysia	229	Dental students	75	154	74.24%	170	Online survey	8 (3.5%)	25 July - 7 August 2022
Winters M, et al. (43)	2022	Cross sectional	United States	856	General population	410	436	46%	394	Online survey	248 (29%)	June 2022
Harapan H, et al. (44)	2020	Cross sectional	Indonesia	407	Physicians	128	279	93.6%	381	Online survey	NR	25 May 2019–25 July 2019

M/F: Male/Female; NR: Not reported; *MSM: men who have sex with men; LGBTI: Lesbian, gay, bisexual, transgender, and intersex

### Quality of the included studies and publication bias

The included cross-sectional studies were characterized by their high level of quality, which was assessed using the JBI-MAStARI tool [16–44] (Table S3). Egger’s test for the evaluation of publication bias obtained a value of p = 0.0005 (t = -3.99, df = 27), thus rejecting the null hypothesis of symmetry. Thus, it can be shown that the asymmetry in the results and in the image explains the wide differences in the reported prevalence values; however, publication bias cannot be demonstrated (Figure S1).

### Prevalence of intention to vaccinate against Mpox

**Fig. 2:**
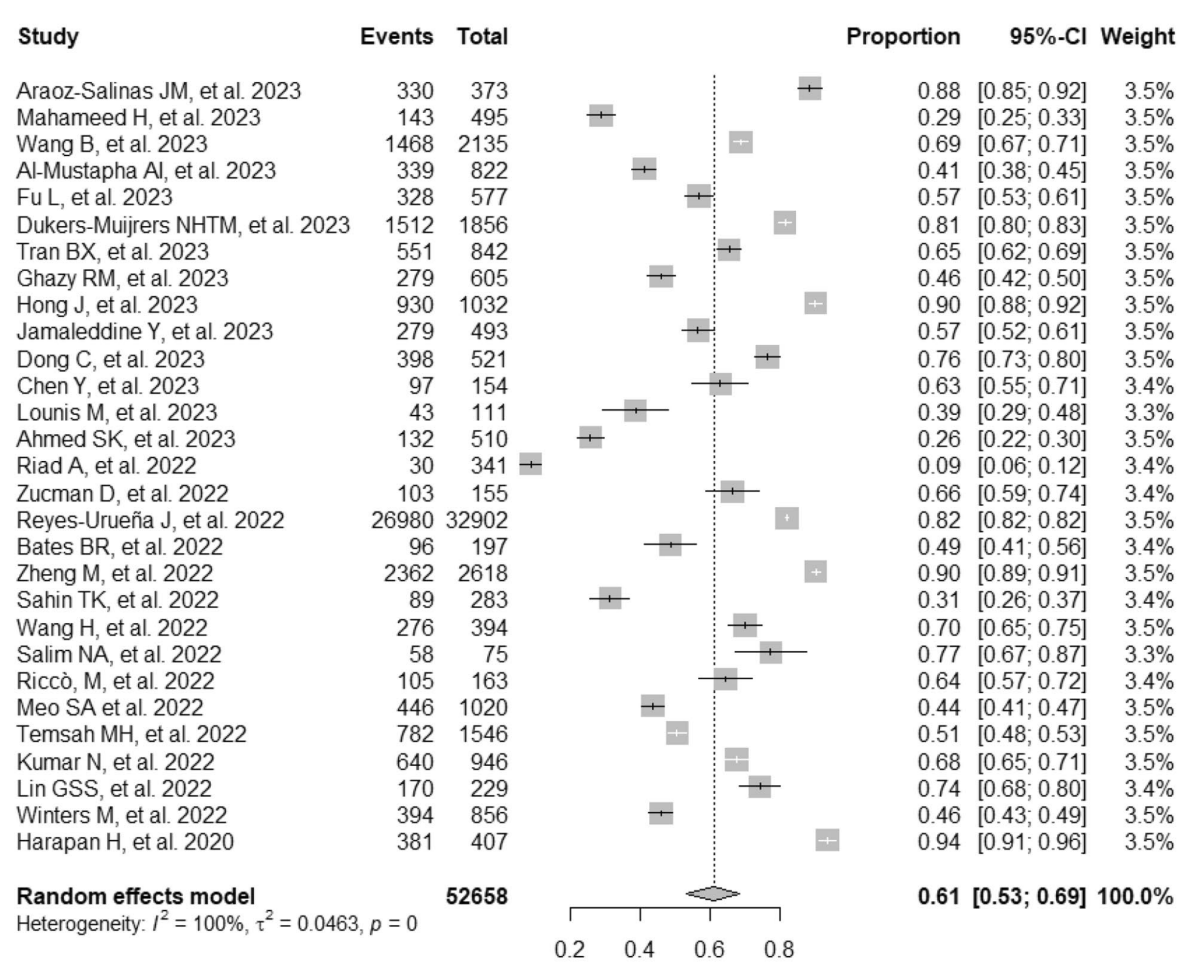
Forest plot illustrating the combined prevalence of intention to vaccinate against monkeypox

**Fig. 3:**
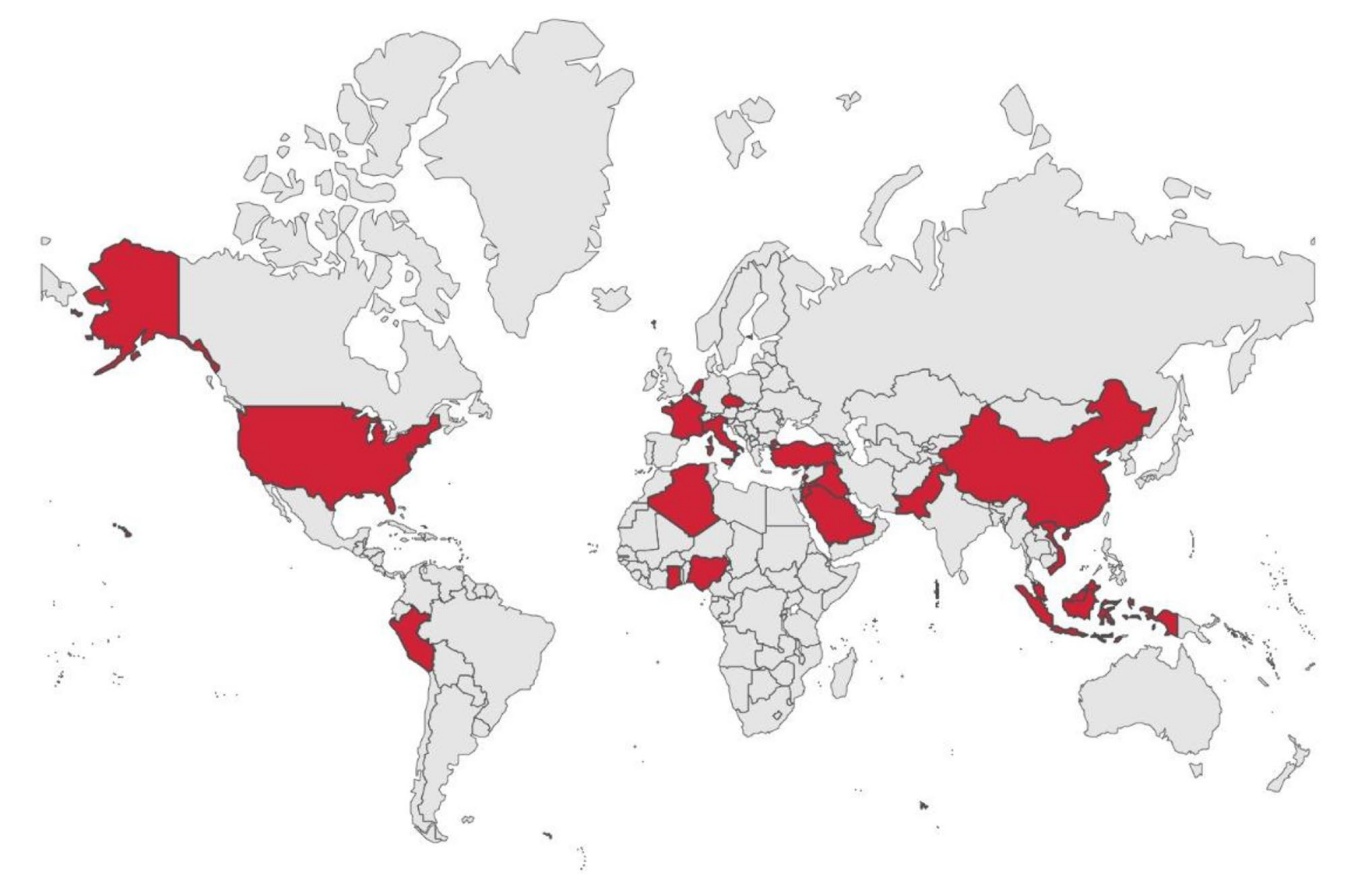
Map illustrating the prevalence of the intention to vaccinate against monkeypox in different countries of the world: Peru (88%), Jordan (29%), China (76%), Nigeria (41%), Netherlands (76%), Vietnam (65%), Ghana (46%), Lebanon (57%), Algeria (39%), Iraq (26%), Czech Republic (9%), France (66%), United States (47%), Turkey (31%), Indonesia (87%), Italy (64%), Saudi Arabia (47%), Pakistan (68%), and Malaysia (74%)

The combined prevalence of the intention to vaccinate against Mpox was 61% (95% CI: 53–69%; 52,658 participants; 29 studies; I^2^ = 100%) [16–44] (Fig. 2). Figure 3 illustrates the pooled prevalence of the intention to vaccinate against Mpox in different countries, according to the data collected in the studies analyzed. Analyzing the data by continent, the following vaccination intention prevalences were found: In Asian countries, it was 64% (95% CI: 53–74%; 13,883 participants; 17 studies; I^2^ = 99%) [17, 18, 20, 22, 24–27, 29, 34, 35, 37, 39–42, 44]; in African countries, it was 43% (95% CI: 39–47%; 1538 participants; 3 studies; I^2^ = 53%) [19, 23, 28]; in European countries, it was 62% (95% CI: 45–78%; 35,811 participants; 6 studies; I^2^ = 99%) [21, 30–32, 36, 38]; and in American countries, it was 63% (95% CI: 32–89%; 1426 participants; 3 studies; I^2^ = 99%) [16, 33, 43] (Figure S3). Furthermore, when focusing on the target population of the studies, the following vaccination intention prevalences against Mpox were observed: among the general population, it was 54% (95% CI: 45–62%; 10,296 participants; 11 studies; I^2^ = 99%) [18, 19, 22, 23, 25, 26, 29, 39–41, 43]; among healthcare workers, it was 57% (95% CI: 33–79%; 3333 participants; 10 studies; I^2^ = 99%) [17, 24, 28, 30, 33, 35, 37, 38, 42, 44]; and among the LGBTI community, it was 76% (95% CI: 70–82%;39,029 participants; 8 studies; I^2^ = 98%) [16, 20, 21, 27, 31, 32, 34, 36] (Figure S5).

### Prevalence of refusal of vaccination against Mpox

The aggregated prevalence of vaccination refusal against Mpox was found to be 22% (95% CI: 16–30%; 45,577 participants; 21 studies; I^2^ = 99%) [16–18, 20–27, 29–33, 35, 37, 41–43] (Figure S2). When analyzing the data by continents, the following prevalence rates of vaccination refusal against Mpox were observed: in Asian countries, 19% (95% CI: 11–28%; 8292 participants; 13 studies; I^2^ = 99%) [17, 18, 20, 22, 24–27, 29, 35, 37, 41, 42]; in European countries, 23% (95% CI: 12–35%; 35,254 participants; 4 studies; I^2^ = 99%) [21, 30–32]; and in American countries, 29% (95% CI: 12–50%;1426 participants; 3 studies; I^2^ = 98%) [16, 33, 43] (Figure S4). Furthermore, a subgroup analysis focused on the target population of the studies was conducted, and the following prevalence rates of vaccination refusal against Mpox were found: among the general population, 22% (95% CI: 11–36%; 6908 participants; 8 studies; I^2^ = 99%) [18, 22, 23, 25, 26, 29, 41, 43]; among healthcare workers, 23% (95% CI: 10–39%; 2652 participants; 7 studies; I^2^ = 99%) [17, 24, 30, 33, 35, 37, 42]; and among the LGBTI community, 22% (95% CI: 13–34%;36,017 participants; 6 studies; I^2^ = 99%) [16, 20, 21, 27, 31, 32] (Figure S6).

## Discussion

Improving vaccination is essential for several diseases with available vaccines. In addition to creating safe and effective vaccines, it is necessary to solve logistical challenges, ensure equitable distribution, and promote acceptance in the population to guarantee the demand for vaccines [45].

Monkeypox is gradually becoming a globally relevant public health issue. There are still uncertainties regarding the exact routes of transmission of this disease [8, 46]. Therefore, it is essential to propose sound preventive approaches, such as the implementation of targeted vaccination programs against the Mpox virus, to address this issue efficiently [45].

The present systematic review and meta-analysis determined the prevalence of intention to receive the Mpox vaccine. The combined prevalence of intention to be vaccinated against Mpox was 61%. According to investigations, the prevalence of intention to be vaccinated against Mpox ranged from 8.8 to 93.6% [30, 44]. Riad A et al. showed that 51% of participants were willing to receive the Mpox vaccine if it was offered free, safe, and effective [47]. Another study proposed by Alarifi AM et al. reported that 52.7% of the participants expressed a willingness to receive the Mpox vaccine. The results indicated that the main reasons for this willingness were trust in the Saudi Arabian Ministry of Health (57.7%) and perception of the vaccine as a social responsibility (44.6%) [48]. A systematic review and meta-analysis study proposed by Ulloque-Badaracco JR et al. reported a pooled prevalence of acceptance of the Mpox vaccination of 56% [45].

Globally, vaccination represents a fundamental strategy to mitigate both the spread and severity of contagious viral infections, especially for immunocompromised individuals [49]. Smallpox vaccination provides cross-protection for both smallpox and Mpox, preventing approximately 85% of Mpox virus infection. Two vaccines are available: modified vaccinia Ankara (Jynneos/Imamune/Imvanex, Bavarian Nordic, Hørsholm, Denmark) and ACAM2000 (Emergent BioSolutions, Gaithersburg, MD, USA) [50, 51].

In the subgroup analysis by continents on the intention to be vaccinated against Mpox, the following prevalences were found: Asia (64%), Europe (62%), America (63%), and Africa (43%). Ulloque-Badaracco JR, et al. reported that the prevalence of Mpox vaccine uptake was 50% in Asian countries and 70% in European countries [45]. In addition, in China and Indonesia, they reported the highest prevalence of intention to vaccinate against Mpox, around 90.2% and 93.6%, respectively [34, 44]. This variation could be due to how different countries respond to the severity of a disease and take precautions, which is related to socioeconomic and cultural factors, access to information, and distrust in the health system and government policies.

In the subgroup analysis on the intention to be vaccinated against Mpox, focused on the target population of the studies, the following prevalences were found: general population (54%), health care workers (57%), and the LGBTI community (76%). The study conducted by Alarifi AM et al. revealed that physicians and pharmacists demonstrated a higher willingness to receive the Mpox vaccine, with percentages of 57.5% and 56.1%, respectively, compared to nurses, whose willingness was 46.7% [48]. Ulloque-Badaracco JR et al. reported that the prevalence of vaccine acceptance was 43.0% in the general population, 63.0% in health care workers, and 84.0% in the LGBTI community [45]. In addition, the results may indicate an increased awareness among study subjects of the importance of prevention in different groups that have faced barriers to medical care. The current Mpox outbreak continues to impact primarily men who have sex with men and who have reported having recent sexual encounters with one or more male partners [52]. Therefore, it is crucial to monitor people who have been in contact with the reported cases in order to prevent the spread of this disease.

Another important secondary outcome found by the study was that the pooled prevalence of Mpox vaccination refusal was 22%. Finally, it is worth mentioning that both Americans and healthcare workers exhibited the highest rates of refusal towards Mpox vaccination, with 29% and 23% refusal, respectively. Riad A et al. showed that 30.6% and 18.1% of participants were unsure and refused the Mpox vaccination [47]. Another study proposed by Alarifi AM et al. reported that 47.3% of participants refused the Mpox vaccination [48]. Ulloque-Badaracco JR et al. in their systematic review and meta-analysis, reported a refusal of Mpox vaccination of 24% [45]. One investigation identified insufficient information about the vaccine, fear of unknown adverse reactions, and doubts about the effectiveness and safety of the vaccine as the most reported reasons for unwillingness to receive the Mpox vaccine [48].

This study highlights the importance of recognizing regional and subgroup disparities in willingness to vaccinate and refusal of Mpox vaccination. The findings emphasize the need to implement communication and education strategies tailored to particular contexts in order to enhance vaccination uptake. Additionally, identifying populations with higher refusal rates can guide specific efforts to address concerns and strengthen vaccine confidence within these groups. Ultimately, understanding these factors is essential to achieving optimal levels of vaccination coverage and safeguarding global public health.

The present study has some limitations. First, information about Mpox is constantly evolving. Second, it is crucial to recognize the possibility of bias in the incorporated studies. Third, it is important to keep in mind that the studies addressed in the meta-analysis may cover diverse populations, interventions, and outcomes, thus making it difficult to extrapolate the findings to other populations. In addition, it is crucial to improve the instruments and methods for measuring the intention, acceptance, and refusal of the Mpox vaccination. Several factors, such as confidence in the efficacy and safety of the vaccine, health professionals’ recommendations, government policies, perceptions of disease risk, as well as other social and cultural aspects, may influence these attitudes. It is suggested that future research should focus on assessing the Mpox vaccine acceptance variable, which is defined as a person’s willingness to receive or adopt a specific vaccine, supported by confidence and safety in that vaccine. Regarding its strengths, this current study has a rigorous methodological approach, as it was conducted following the guidelines proposed by the PRISMA guidelines. Furthermore, it constitutes the first systematic review and meta-analysis analyzing the prevalence of the intention to receive the Mpox vaccine. In addition, all the procedures used to select the studies were performed independently by two or more authors.

## Conclusions

A combined prevalence of 61% of the intention to vaccinate against Mpox was found, with significant differences across continents and the target population of the studies. Additionally, a considerable prevalence of vaccination refusals against Mpox was identified in different groups and regions, highlighting the importance of implementing appropriate strategies to enhance vaccination acceptance and understanding.

### Electronic supplementary material

Below is the link to the electronic supplementary material.

Supplementary Material 1

## Data Availability

All data generated or analyzed during this study are included in this published article.
