# Supplementary material for: Prevalence of intentions to receive monkeypox vaccine. A systematic review and meta-analysis
Source: BMC Public Health. 2024 Jan 2;24:35. doi: 10.1186/s12889-023-17473-y (PMC10763398; doi:10.1186/s12889-023-17473-y)
Supplement: Supplementary file 1 — Supplementary Material 1 [file 12889_2023_17473_MOESM1_ESM.docx]

Systematic Review

Prevalence of intentions to receive monkeypox vaccine. A systematic review and meta-analysis.

Darwin A. León-Figueroa ^1^, Joshuan J. Barboza ^2^, Mario J. Valladares-Garrido ^3,4,^*, Ranjit Sah ^5,6,7^, and Alfonso J. Rodriguez-Morales ^8,9^

^1^ Facultad de Medicina Humana, Universidad de San Martín de Porres, Chiclayo 15011, Peru; [darwin_leon@usmp.pe](mailto:darwin_leon@usmp.pe) (DALF)

^2^ Vicerrectorado de Investigación, Universidad Norbert Wiener, Lima 15046, Peru; [jbarbozameca@gmail.com](mailto:jbarbozameca@gmail.com)

^3^ Universidad Continental, Lima 15046, Peru; [mvalladares@continental.edu.pe](mailto:mvalladares@continental.edu.pe)

^4^ Oficina de Epidemiología, Hospital Regional Lambayeque, Chiclayo 14012, Peru

^5^ Department of Microbiology, Tribhuvan University Teaching Hospital, Institute of Medicine, Kathmandu 44600, Nepal; [ranjitsah@iom.edu.np](mailto:ranjitsah@iom.edu.np)

^6^ Department of Microbiology, Dr. D. Y. Patil Medical College, Hospital and Research Centre, Dr. D. Y. Patil Vidyapeeth, Pune 411018, Maharashtra, India

^7^ Department of Public Health Dentistry, Dr. D.Y. Patil Dental College and Hospital, Dr. D.Y. Patil Vidyapeeth, Pune 411018, Maharashtra, India.

^8^ Master of Clinical Epidemiology and Biostatistics, Universidad Cientifica del Sur, Lima 15067, Peru; [arodriguezmo@cientifica.edu.pe](mailto:arodriguezmo@cientifica.edu.pe)

^9^ Gilbert and Rose-Marie Chagoury School of Medicine, Lebanese American University, Beirut 1102, Lebanon; [alphonso.morales@lau.edu.lb](mailto:alphonso.morales@lau.edu.lb)

***** **Correspondence**: Correspondence: [mvalladares@continental.edu.pe](mailto:mvalladares@continental.edu.pe) ; Tel.: +51 944 655 396

**Supplementary material**

- **Table S1. PRISMA Checklist** (PRISMA 2020 Main Checklist and PRIMSA Abstract Checklist)
- **Table S2.** The adjusted search terms as per searched electronic databases.
- **Table S3.** Quality of the included studies
- **Figure S1**: Funnel plot and Egger's test illustrate the publication bias of the included studies.
- **Figure S2**: Forest plot illustrating the combined prevalence of vaccine refusal against monkeypox.
- **Figure S3.** Forest plot illustrating the prevalence of monkeypox vaccination intention among according to continents.
- **Figure S4.** Forest plot illustrating the prevalence of monkeypox vaccination rejection among according to continents.
- **Figure S5**. Forest plot illustrating the prevalence of monkeypox vaccination intention among study subjects.
- **Figure S6**. Forest plot illustrating the prevalence of monkeypox vaccination rejection among study subjects.
- **References**

**Table S1. PRISMA Checklist** (PRISMA 2020 Main Checklist and PRIMSA Abstract Checklist)

# PRISMA 2020 Main Checklist

| **Topic** | **No.** | **Item** | **Location where item is reported** |
| --- | --- | --- | --- |
| **TITLE** |  |  |  |
| **Title** | 1 | Identify the report as a systematic review. | 1 |
| **ABSTRACT** |  |  |  |
| **Abstract** | 2 | See the PRISMA 2020 for Abstracts checklist |  |
| **INTRODUCTION** |  |  |  |
| **Rationale** | 3 | Describe the rationale for the review in the context of existing knowledge. | 2 |
| **Objectives** | 4 | Provide an explicit statement of the objective(s) or question(s) the review addresses. | 2-3 |
| **METHODS** |  |  |  |
| **Eligibility criteria** | 5 | Specify the inclusion and exclusion criteria for the review and how studies were grouped for the syntheses. | 3 |
| **Information sources** | 6 | Specify all databases, registers, websites, organisations, reference lists and other sources searched or consulted to identify studies. Specify the date when each source was last searched or consulted. | 3 |
| **Search strategy** | 7 | Present the full search strategies for all databases, registers and websites, including any filters and limits used. | 3 |
| **Selection process** | 8 | Specify the methods used to decide whether a study met the inclusion criteria of the review, including how many reviewers screened each record and each report retrieved, whether they worked independently, and if applicable, details of automation tools used in the process. | 3 |
| **Data collection process** | 9 | Specify the methods used to collect data from reports, including how many reviewers collected data from each report, whether they worked independently, any processes for obtaining or confirming data from study investigators, and if applicable, details of automation tools used in the process. | 4 |
| **Data items** | 10a | List and define all outcomes for which data were sought. Specify whether all results that were compatible with each outcome domain in each study were sought (e.g. for all measures, time points, analyses), and if not, the methods used to decide which results to collect. | 4 |
|  | 10b | List and define all other variables for which data were sought (e.g. participant and intervention characteristics, funding sources). Describe any assumptions made about any missing or unclear information. | 4 |
| **Study risk of bias assessment** | 11 | Specify the methods used to assess risk of bias in the included studies, including details of the tool(s) used, how many reviewers assessed each study and whether they worked independently, and if applicable, details of automation tools used in the process. | 4 |
| **Effect measures** | 12 | Specify for each outcome the effect measure(s) (e.g. risk ratio, mean difference) used in the synthesis or presentation of results. | 4 |
| **Synthesis methods** | 13a | Describe the processes used to decide which studies were eligible for each synthesis (e.g. tabulating the study intervention characteristics and comparing against the planned groups for each synthesis (item 5)). | 4 |
|  | 13b | Describe any methods required to prepare the data for presentation or synthesis, such as handling of missing summary statistics, or data conversions. | 4 |
|  | 13c | Describe any methods used to tabulate or visually display results of individual studies and syntheses. | 4 |
|  | 13d | Describe any methods used to synthesize results and provide a rationale for the choice(s). If meta-analysis was performed, describe the model(s), method(s) to identify the presence and extent of statistical heterogeneity, and software package(s) used. | 4 |
|  | 13e | Describe any methods used to explore possible causes of heterogeneity among study results (e.g. subgroup analysis, meta-regression). | 4 |
|  | 13f | Describe any sensitivity analyses conducted to assess robustness of the synthesized results. | 4 |
| **Reporting bias assessment** | 14 | Describe any methods used to assess risk of bias due to missing results in a synthesis (arising from reporting biases). | 4 |
| **Certainty assessment** | 15 | Describe any methods used to assess certainty (or confidence) in the body of evidence for an outcome. | 4 |
| **RESULTS** |  |  |  |
| **Study selection** | 16a | Describe the results of the search and selection process, from the number of records identified in the search to the number of studies included in the review, ideally using a flow diagram. | 4 |
|  | 16b | Cite studies that might appear to meet the inclusion criteria, but which were excluded, and explain why they were excluded. | 4 |
| **Study characteristics** | 17 | Cite each included study and present its characteristics. | 5 |
| **Risk of bias in studies** | 18 | Present assessments of risk of bias for each included study. | 5 |
| **Results of individual studies** | 19 | For all outcomes, present, for each study: (a) summary statistics for each group (where appropriate) and (b) an effect estimate and its precision (e.g. confidence/credible interval), ideally using structured tables or plots. | 5 |
| **Results of syntheses** | 20a | For each synthesis, briefly summarise the characteristics and risk of bias among contributing studies. | 5 |
|  | 20b | Present results of all statistical syntheses conducted. If meta-analysis was done, present for each the summary estimate and its precision (e.g. confidence/credible interval) and measures of statistical heterogeneity. If comparing groups, describe the direction of the effect. | 5 |
|  | 20c | Present results of all investigations of possible causes of heterogeneity among study results. | 5 |
|  | 20d | Present results of all sensitivity analyses conducted to assess the robustness of the synthesized results. | 5 |
| **Reporting biases** | 21 | Present assessments of risk of bias due to missing results (arising from reporting biases) for each synthesis assessed. | 5 |
| **Certainty of evidence** | 22 | Present assessments of certainty (or confidence) in the body of evidence for each outcome assessed. | 5 |
| **DISCUSSION** |  |  |  |
| **Discussion** | 23a | Provide a general interpretation of the results in the context of other evidence. | 6 |
|  | 23b | Discuss any limitations of the evidence included in the review. | 6 |
|  | 23c | Discuss any limitations of the review processes used. | 7 |
|  | 23d | Discuss implications of the results for practice, policy, and future research. | 7 |
| **OTHER INFORMATION** |  |  |  |
| **Registration and protocol** | 24a | Provide registration information for the review, including register name and registration number, or state that the review was not registered. | 3 |
|  | 24b | Indicate where the review protocol can be accessed, or state that a protocol was not prepared. | 3 |
|  | 24c | Describe and explain any amendments to information provided at registration or in the protocol. | 3 |
| **Support** | 25 | Describe sources of financial or non-financial support for the review, and the role of the funders or sponsors in the review. | 7 |
| **Competing interests** | 26 | Declare any competing interests of review authors. | 7 |
| **Availability of data, code and other materials** | 27 | Report which of the following are publicly available and where they can be found: template data collection forms; data extracted from included studies; data used for all analyses; analytic code; any other materials used in the review. | 7 |

#####

# PRIMSA Abstract Checklist

| **Topic** | **No.** | **Item** | **Reported?** |
| --- | --- | --- | --- |
| **TITLE** |  |  |  |
| **Title** | 1 | Identify the report as a systematic review. | Yes |
| **BACKGROUND** |  |  |  |
| **Objectives** | 2 | Provide an explicit statement of the main objective(s) or question(s) the review addresses. | Yes |
| **METHODS** |  |  |  |
| **Eligibility criteria** | 3 | Specify the inclusion and exclusion criteria for the review. | Yes |
| **Information sources** | 4 | Specify the information sources (e.g. databases, registers) used to identify studies and the date when each was last searched. | Yes |
| **Risk of bias** | 5 | Specify the methods used to assess risk of bias in the included studies. | Yes |
| **Synthesis of results** | 6 | Specify the methods used to present and synthesize results. | Yes |
| **RESULTS** |  |  |  |
| **Included studies** | 7 | Give the total number of included studies and participants and summarise relevant characteristics of studies. | Yes |
| **Synthesis of results** | 8 | Present results for main outcomes, preferably indicating the number of included studies and participants for each. If meta-analysis was done, report the summary estimate and confidence/credible interval. If comparing groups, indicate the direction of the effect (i.e. which group is favoured). | Yes |
| **DISCUSSION** |  |  |  |
| **Limitations of evidence** | 9 | Provide a brief summary of the limitations of the evidence included in the review (e.g. study risk of bias, inconsistency and imprecision). | Yes |
| **Interpretation** | 10 | Provide a general interpretation of the results and important implications. | Yes |
| **OTHER** |  |  |  |
| **Funding** | 11 | Specify the primary source of funding for the review. | Yes |
| **Registration** | 12 | Provide the register name and registration number. | Yes |

*From:* Page MJ, McKenzie JE, Bossuyt PM, Boutron I, Hoffmann TC, Mulrow CD, et al. The PRISMA 2020 statement: an updated guideline for reporting systematic reviews. MetaArXiv. 2020, September 14. DOI: 10.31222/osf.io/v7gm2. For more information, visit: <www.prisma-statement.org>

**Table S2.** The adjusted search terms as per searched electronic databases.

| **PubMed** | | | |
| --- | --- | --- | --- |
|  | #1 | (Monkeypox OR “Monkey Pox” OR “Mpox”) | 3,689 |
|  | #2 | (“ Attitude” OR “ Attitudes” OR “vaccine”) | 791,278 |
|  | #3 | #1 AND #2 | 690 |
| **Scopus** | | | |
|  | #1 | TITLE-ABS-KEY (Monkeypox OR “Monkey Pox” OR “Mpox”) | 4,302 |
|  | #2 | TITLE-ABS-KEY ( " Attitude" OR " Attitudes" OR “vaccine”) | 1,570,474 |
|  | #3 | #1 AND #2 | 1,327 |
| **Embase** | | | |
|  | #1 | 'monkeypox'/exp OR 'monkeypox' | 4,340 |
|  | #2 | 'attitude' OR 'vaccine' | 1,082,652 |
|  | #3 | #1 AND #2 | 1,384 |
| **Web of Science** | | | |
|  | #1 | ALL=(Monkeypox OR “Monkey Pox” OR “Mpox”) | 4,759 |
|  | #2 | ALL= (“ Attitude” OR “ Attitudes” OR “vaccine”) | 1,203,456 |
|  | #3 | #1 AND #2 | 1,486 |
| **ScienceDirect** | | | |
|  | #1 | (Monkeypox OR “Monkey Pox” OR “Mpox”) | 1,003 |
|  | #2 | (“ Attitude” OR “vaccine”) | 68,951 |
|  | #3 | #1 AND #2 | 63 |

**Table S3**. Quality of the included studies

| Authors | Year | Eligibility criteria | Study subjects and the setting | Exposure measured in a valid and reliable way 'gold standard' | A specified diagnosis or definition | Confounding factors | Dealing with confounding factors | Outcomes measured in a valid and reliable way | Appropriate statistical analysis | Scores (8) | Quality (high, moderate, low) |  |
| --- | --- | --- | --- | --- | --- | --- | --- | --- | --- | --- | --- | --- |
| Araoz-Salinas JM, et al. [1] | | 2023 | Yes | Yes | Yes | Yes | No | NA | Yes | Yes | 7 | High |
| Mahameed H, et al. [2] | | 2023 | Yes | Yes | Yes | Yes | No | NA | Yes | Yes | 7 | High |
| Wang B, et al. [3] | | 2023 | Yes | Yes | Yes | Yes | No | NA | Yes | Yes | 7 | High |
| Al-Mustapha AI, et al. [4] | | 2023 | Yes | Yes | Yes | Yes | No | NA | Yes | Yes | 7 | High |
| Fu L, et al. [5] | | 2023 | Yes | Yes | Yes | Yes | No | NA | Yes | Yes | 7 | High |
| Dukers-Muijrers NHTM, et al. [6] | | 2023 | Yes | Yes | Yes | Yes | No | NA | Yes | Yes | 7 | High |
| Tran BX, et al. [7] | | 2023 | Yes | Yes | Yes | Yes | No | NA | Yes | Yes | 7 | High |
| Ghazy RM, et al. [8] | | 2023 | Yes | Yes | Yes | Yes | No | NA | Yes | Yes | 7 | High |
| Hong J, et al. [9] | | 2023 | Yes | Yes | Yes | Yes | No | NA | Yes | Yes | 7 | High |
| Jamaleddine Y, et al. [10] | | 2023 | Yes | Yes | Yes | Yes | No | NA | Yes | Yes | 7 | High |
| Dong C, et al. [11] | | 2023 | Yes | Yes | Yes | Yes | No | NA | Yes | Yes | 7 | High |
| Chen Y, et al. [12] | | 2023 | Yes | Yes | Yes | Yes | No | NA | Yes | Yes | 7 | High |
| Lounis M, et al. [13] | | 2023 | Yes | Yes | Yes | Yes | No | NA | Yes | Yes | 7 | High |
| Ahmed SK, et al. [14] | | 2023 | Yes | Yes | Yes | Yes | No | NA | Yes | Yes | 7 | High |
| Riad A, et al. [15] | | 2022 | Yes | Yes | Yes | Yes | No | NA | Yes | Yes | 7 | High |
| Zucman D, et al. [16] | | 2022 | Yes | Yes | Yes | Yes | No | NA | Yes | Yes | 7 | High |
| Reyes-Urueña J, et al. [17] | | 2022 | Yes | Yes | Yes | Yes | No | NA | Yes | Yes | 7 | High |
| Bates BR, et al. [18] | | 2022 | Yes | Yes | Yes | Yes | No | NA | Yes | Yes | 7 | High |
| Zheng M, et al. [19] | | 2022 | Yes | Yes | Yes | Yes | No | NA | Yes | Yes | 7 | High |
| Sahin TK, et al. [20] | | 2022 | Yes | Yes | Yes | Yes | No | NA | Yes | Yes | 7 | High |
| Wang H, et al. [21] | | 2022 | Yes | Yes | Yes | Yes | No | NA | Yes | Yes | 7 | High |
| Salim NA, et al. [22] | | 2022 | Yes | Yes | Yes | Yes | No | NA | Yes | Yes | 7 | High |
| Riccò, M, et al. [23] | | 2022 | Yes | Yes | Yes | Yes | No | NA | Yes | Yes | 7 | High |
| Meo SA et al. [24] | | 2022 | Yes | Yes | Yes | Yes | No | NA | Yes | Yes | 7 | High |
| Temsah MH, et al. [25] | | 2022 | Yes | Yes | Yes | Yes | No | NA | Yes | Yes | 7 | High |
| Kumar N, et al. [26] | | 2022 | Yes | Yes | Yes | Yes | No | NA | Yes | Yes | 7 | High |
| Lin GSS, et al. [27] | | 2022 | Yes | Yes | Yes | Yes | No | NA | Yes | Yes | 7 | High |
| Winters M, et al. [28] | | 2022 | Yes | Yes | Yes | Yes | No | NA | Yes | Yes | 7 | High |
| Harapan H, et al. [29] | | 2020 | Yes | Yes | Yes | Yes | No | NA | Yes | Yes | 7 | High |

NA: Not assessed


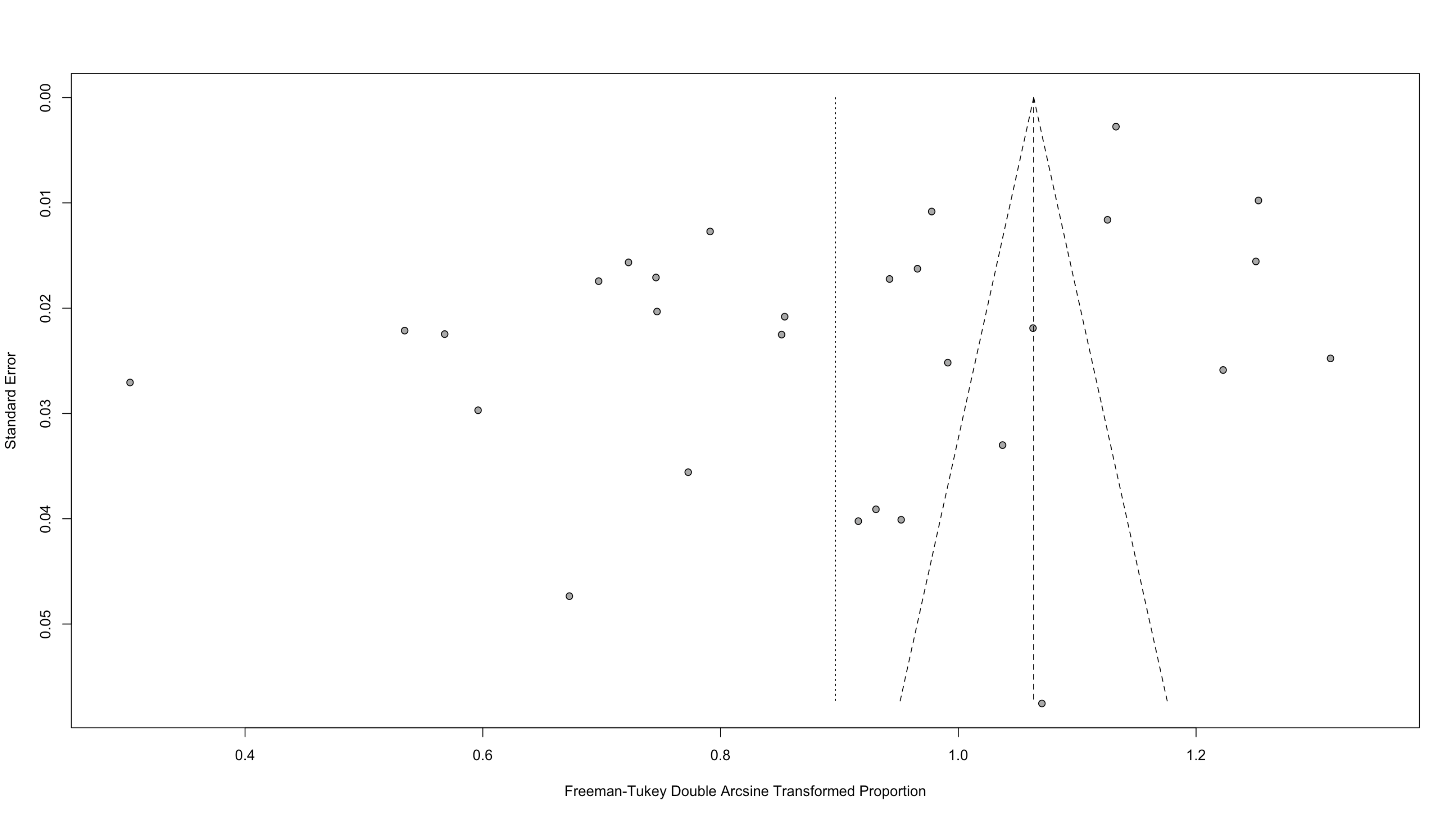

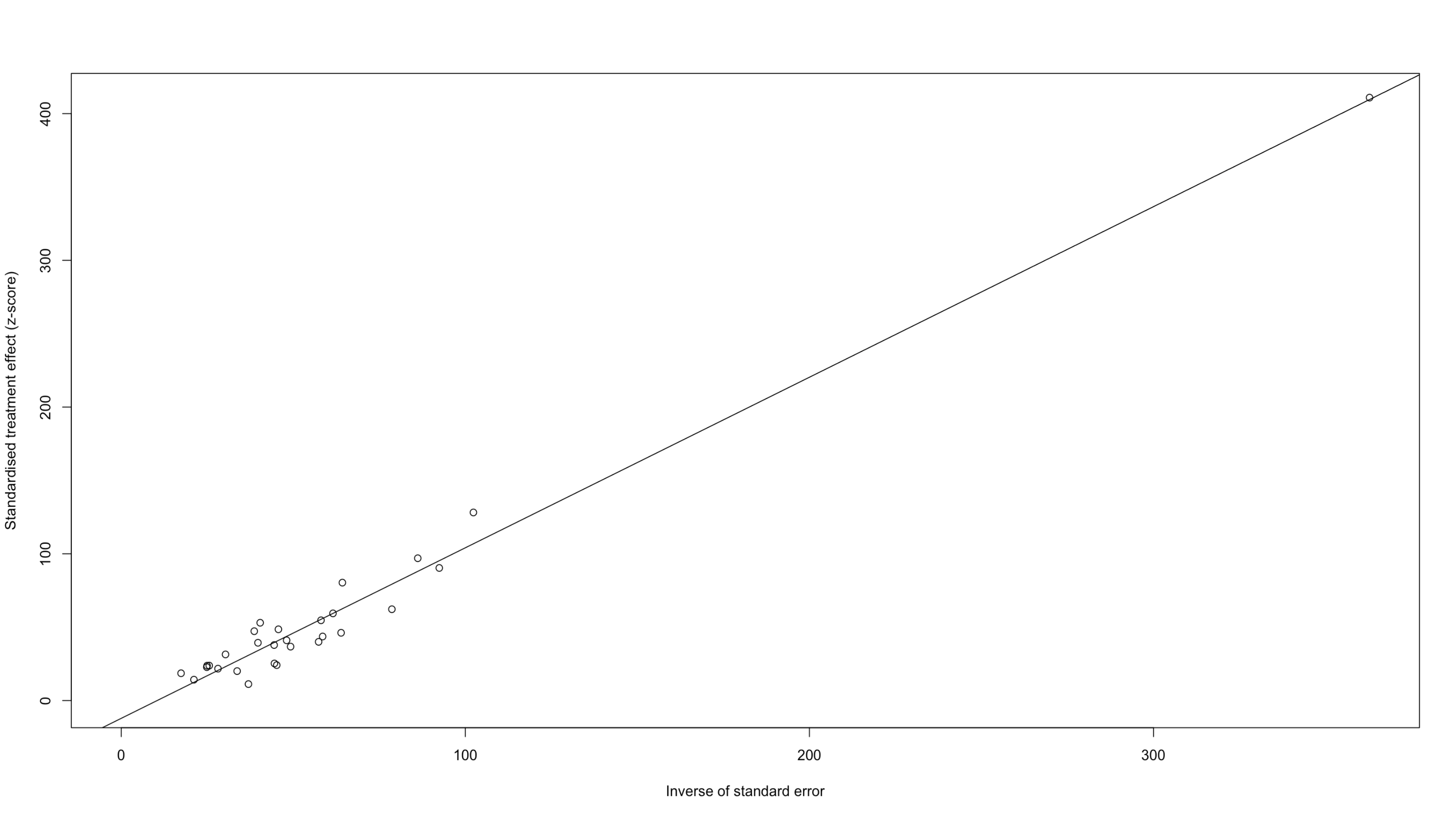


**Figure S1**: Funnel plot and Egger's test illustrate the publication bias of the included studies.

**
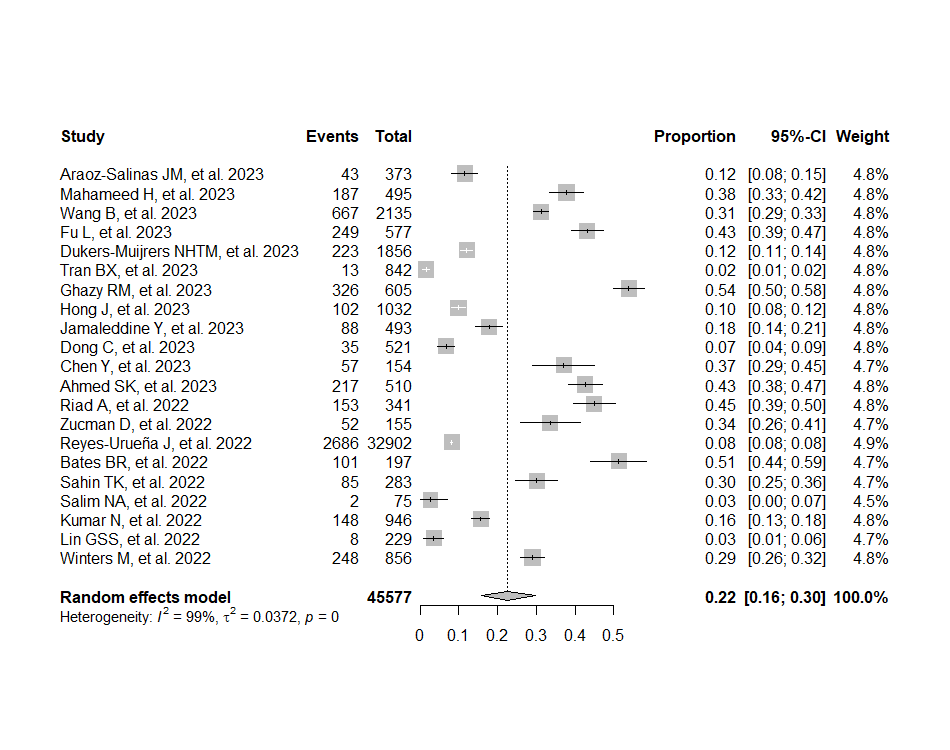
**

**Figure S2**: Forest plot illustrating the combined prevalence of vaccine refusal against monkeypox.


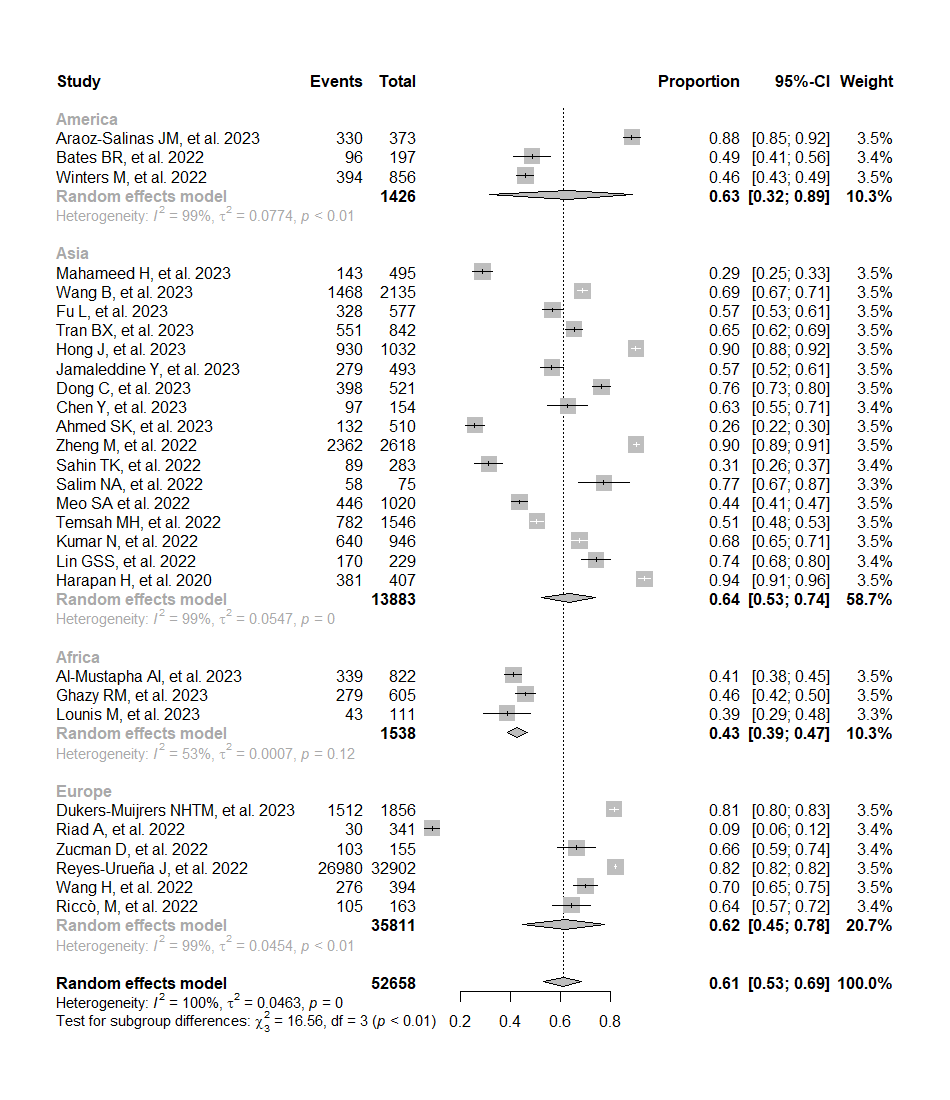


**Figure S3.** Forest plot illustrating the prevalence of monkeypox vaccination intention among according to continents.


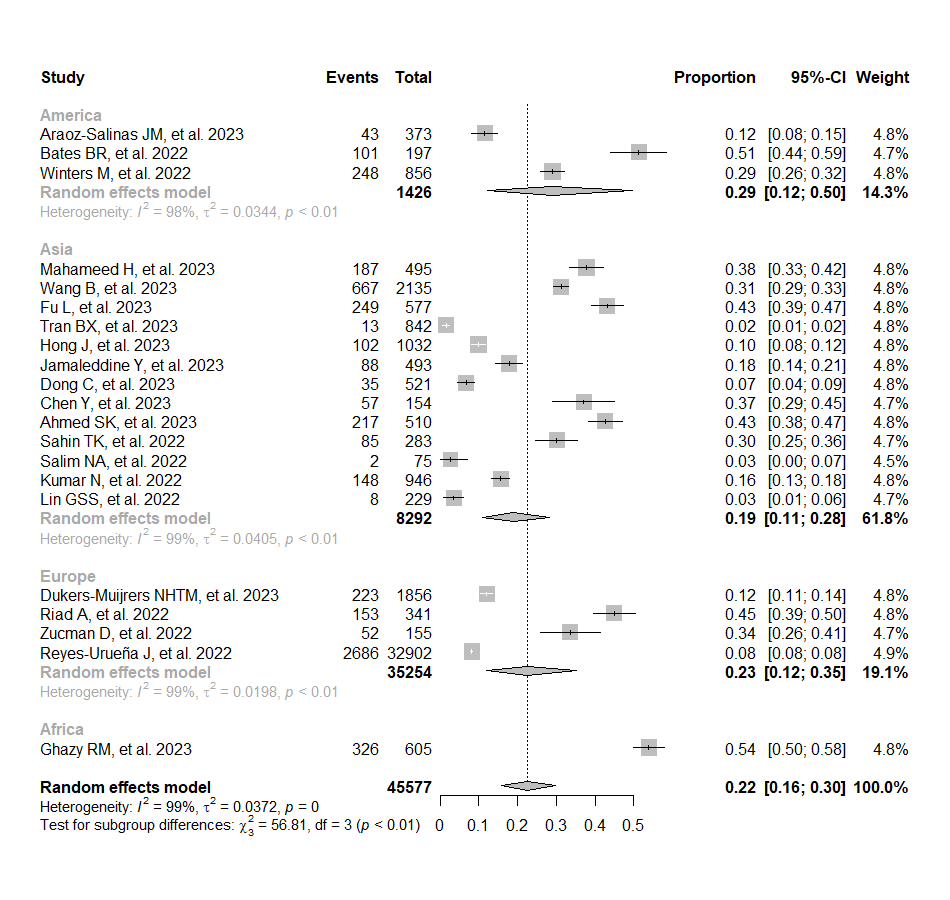


**Figure S4.** Forest plot illustrating the prevalence of monkeypox vaccination rejection among according to continents.


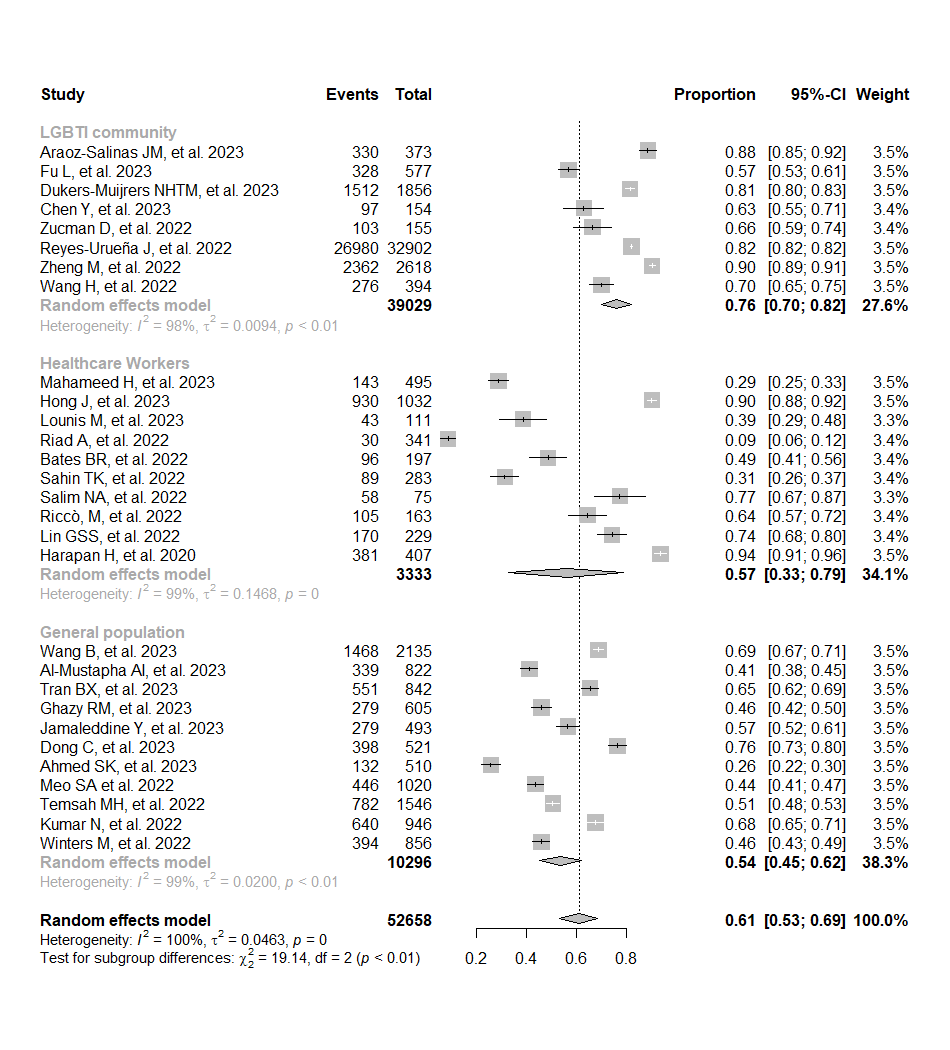


**Figure S5**. Forest plot illustrating the prevalence of monkeypox vaccination intention among study subjects.

**
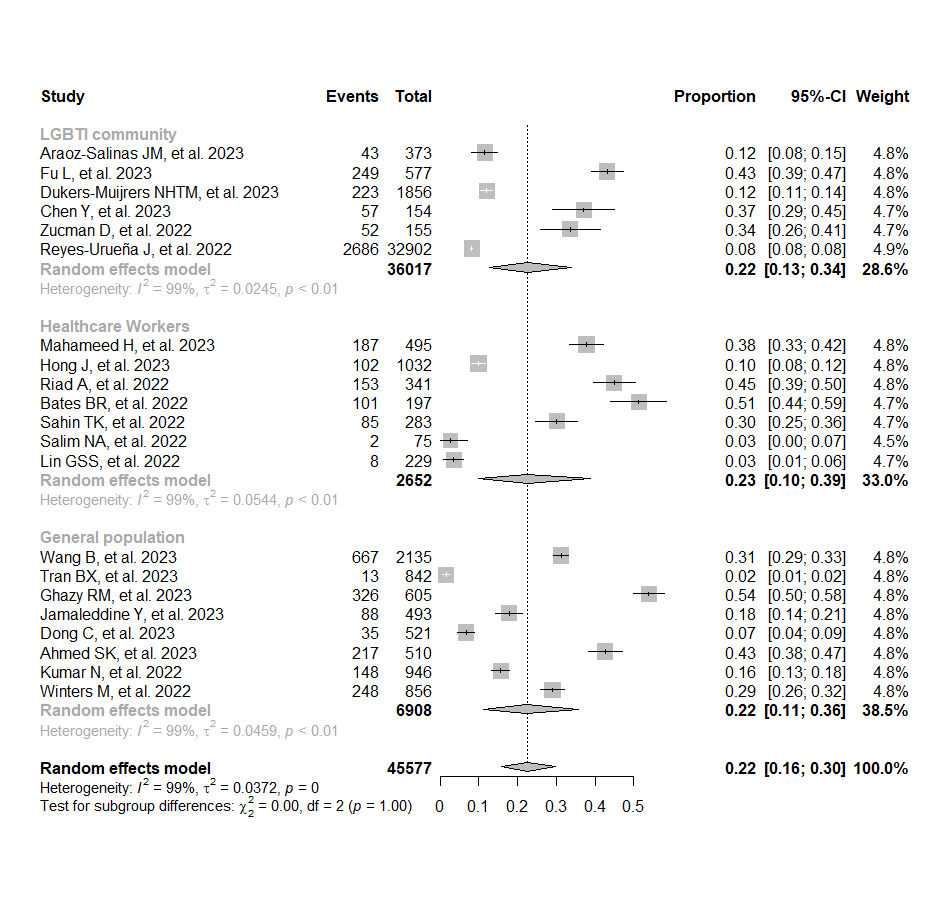
**

**Figure S6**. Forest plot illustrating the prevalence of monkeypox vaccination rejection among study subjects.

References

1. Araoz-Salinas, J.M.; Ortiz-Saavedra, B.; Ponce-Rosas, L.; Soriano-Moreno, D.R.; Soriano-Moreno, A.N.; Alave, J.; Gonzales-Zamora, J.A. Perceptions and Intention to Get Vaccinated against Mpox among the LGBTIQ+ Community during the 2022 Outbreak: A Cross-Sectional Study in Peru. *Vaccines* **2023**, *11*, 1008, doi:10.3390/vaccines11051008.

2. Mahameed, H.; Al-Mahzoum, K.; AlRaie, L.A.; Aburumman, R.; Al-Naimat, H.; Alhiary, S.; Barakat, M.; Al-Tammemi, A.B.; Salim, N.A.; Sallam, M. Previous Vaccination History and Psychological Factors as Significant Predictors of Willingness to Receive Mpox Vaccination and a Favorable Attitude towards Compulsory Vaccination. *Vaccines* **2023**, *11*, 897, doi:10.3390/vaccines11050897.

3. Wang, B.; Peng, X.; Li, Y.; Fu, L.; Tian, T.; Liang, B.; Sun, Y.; Chen, Y.; Wu, X.; Liu, Q.; et al. Perceptions, Precautions, and Vaccine Acceptance Related to Monkeypox in the Public in China: A Cross-Sectional Survey. *J. Infect. Public Health* **2023**, *16*, 163–170, doi:10.1016/j.jiph.2022.12.010.

4. Al-Mustapha, A.I.; Ogundijo, O.A.; Sikiru, N.A.; Kolawole, B.; Oyewo, M.; El-Nadi, H.; Mustapha, A.M.; Adebudo, L.I.; Odukoya, A.; Asiegbu, E.C.; et al. A Cross-Sectional Survey of Public Knowledge of the Monkeypox Disease in Nigeria. *BMC Public Health* **2023**, *23*, 591, doi:10.1186/s12889-023-15398-0.

5. Fu, L.; Sun, Y.; Li, Y.; Wang, B.; Yang, L.; Tian, T.; Wu, X.; Peng, X.; Liu, Q.; Chen, Y.; et al. Perception of and Vaccine Readiness towards Mpox among Men Who Have Sex with Men Living with HIV in China: A Cross-Sectional Study. *Vaccines* **2023**, *11*, 528, doi:10.3390/vaccines11030528.

6. Dukers-Muijrers, N.H.T.M.; Evers, Y.; Widdershoven, V.; Davidovich, U.; Adam, P.C.G.; Op de Coul, E.L.M.; Zantkuijl, P.; Matser, A.; Prins, M.; de Vries, H.J.C.; et al. Mpox Vaccination Willingness, Determinants, and Communication Needs in Gay, Bisexual, and Other Men Who Have Sex with Men, in the Context of Limited Vaccine Availability in the Netherlands (Dutch Mpox-Survey). *Front. Public Health* **2022**, *10*, 1058807, doi:10.3389/fpubh.2022.1058807.

7. Tran, B.X.; Anh Do, L.; Hoang, T.P.; Boyer, L.; Auquier, P.; Fond, G.; Le, H.T.; Le Vu, M.N.; Dang, T.H.T.; Nguyen, A.H.T.; et al. Crucial Choices in a Global Health Crisis: Revealing the Demand and Willingness to Pay for a Hypothetical Monkeypox Vaccine - the PREVENT Study. *J. Glob. Health* **2023**, *13*, 04033, doi:10.7189/jogh.13.04033.

8. Ghazy, R.M.; Yazbek, S.; Gebreal, A.; Hussein, M.; Addai, S.A.; Mensah, E.; Sarfo, M.; Kofi, A.; Al-Ahdal, T.; Eshun, G. Monkeypox Vaccine Acceptance among Ghanaians: A Call for Action. *Vaccines* **2023**, *11*, 240, doi:10.3390/vaccines11020240.

9. Hong, J.; Pan, B.; Jiang, H.-J.; Zhang, Q.-M.; Xu, X.-W.; Jiang, H.; Ye, J.-E.; Cui, Y.; Yan, X.-J.; Zhai, X.-F.; et al. The Willingness of Chinese Healthcare Workers to Receive Monkeypox Vaccine and Its Independent Predictors: A Cross-Sectional Survey. *J. Med. Virol.* **2023**, *95*, e28294, doi:10.1002/jmv.28294.

10. Jamaleddine, Y.; El Ezz, A.A.; Mahmoud, M.; Ismail, O.; Saifan, A.; Mayta, Z.; Saab, A.; Salameh, P. Knowledge and Attitude towards Monkeypox among the Lebanese Population and Their Attitude towards Vaccination. *J. Prev. Med. Hyg.* **2023**, *64*, E13–E26, doi:10.15167/2421-4248/jpmh2023.64.1.2903.

11. Dong, C.; Yu, Z.; Zhao, Y.; Ma, X. Knowledge and Vaccination Intention of Monkeypox in China’s General Population: A Cross-Sectional Online Survey. *Travel Med. Infect. Dis.* **2023**, *52*, 102533, doi:10.1016/j.tmaid.2022.102533.

12. Chen, Y.; Li, Y.; Fu, L.; Zhou, X.; Wu, X.; Wang, B.; Peng, X.; Sun, Y.; Liu, Q.; Lin, Y.-F.; et al. Knowledge of Human Mpox (Monkeypox) and Attitude towards Mpox Vaccination among Male Sex Workers in China: A Cross-Sectional Study. *Vaccines* **2023**, *11*, 285, doi:10.3390/vaccines11020285.

13. Lounis, M.; Bencherit, D.; Abdelhadi, S. Knowledge and Awareness of Algerian Healthcare Workers about Human Monkeypox and Their Attitude toward Its Vaccination: An Online Cross-Sectional Survey. *Vacunas* **2023**, *24*, 122–127, doi:10.1016/j.vacun.2022.11.003.

14. Ahmed, S.K.; Abdulqadir, S.O.; Omar, R.M.; Abdullah, A.J.; Rahman, H.A.; Hussein, S.H.; Mohammed Amin, H.I.; Chandran, D.; Sharma, A.K.; Dhama, K.; et al. Knowledge, Attitude and Worry in the Kurdistan Region of Iraq during the Mpox (Monkeypox) Outbreak in 2022: An Online Cross-Sectional Study. *Vaccines* **2023**, *11*, 610, doi:10.3390/vaccines11030610.

15. Riad, A.; Drobov, A.; Rozmarinová, J.; Drapáčová, P.; Klugarová, J.; Dušek, L.; Pokorná, A.; Klugar, M. Monkeypox Knowledge and Vaccine Hesitancy of Czech Healthcare Workers: A Health Belief Model (HBM)-Based Study. *Vaccines* **2022**, *10*, 2022, doi:10.3390/vaccines10122022.

16. Zucman, D.; Fourn, E.; Touche, P.; Majerholc, C.; Vallée, A. Monkeypox Vaccine Hesitancy in French Men Having Sex with Men with PrEP or Living with HIV in France. *Vaccines* **2022**, *10*, 1629, doi:10.3390/vaccines10101629.

17. Reyes-Urueña, J.; D’Ambrosio, A.; Croci, R.; Bluemel, B.; Cenciarelli, O.; Pharris, A.; Dukers-Muijrers, N.; Nutland, W.; Niaupari, S.; Badran, J.; et al. High Monkeypox Vaccine Acceptance among Male Users of Smartphone-Based Online Gay-Dating Apps in Europe, 30 July to 12 August 2022. *Euro Surveill. Bull. Eur. Sur Mal. Transm. Eur. Commun. Dis. Bull.* **2022**, *27*, 2200757, doi:10.2807/1560-7917.ES.2022.27.42.2200757.

18. Bates, B.R.; Grijalva, M.J. Knowledge, Attitudes, and Practices towards Monkeypox during the 2022 Outbreak: An Online Cross-Sectional Survey among Clinicians in Ohio, USA. *J. Infect. Public Health* **2022**, *15*, 1459–1465, doi:10.1016/j.jiph.2022.11.004.

19. Zheng, M.; Qin, C.; Qian, X.; Yao, Y.; Liu, J.; Yuan, Z.; Ma, L.; Fan, J.; Tao, R.; Zhou, F.; et al. Knowledge and Vaccination Acceptance toward the Human Monkeypox among Men Who Have Sex with Men in China. *Front. Public Health* **2022**, *10*, 997637, doi:10.3389/fpubh.2022.997637.

20. Sahin, T.K.; Erul, E.; Aksun, M.S.; Sonmezer, M.C.; Unal, S.; Akova, M. Knowledge and Attitudes of Turkish Physicians towards Human Monkeypox Disease and Related Vaccination: A Cross-Sectional Study. *Vaccines* **2022**, *11*, 19, doi:10.3390/vaccines11010019.

21. Wang, H.; Paulo, K.J.I. d’Abreu de; Gültzow, T.; Zimmermann, H.M.L.; Jonas, K.J. Monkeypox Self-Diagnosis Abilities, Determinants of Vaccination and Self-Isolation Intention after Diagnosis among MSM, the Netherlands, July 2022. *Eurosurveillance* **2022**, *27*, 2200603, doi:10.2807/1560-7917.ES.2022.27.33.2200603.

22. Salim, N.A.; Septadina, I.S.; Permata, M.; Hudari, H. KNOWLEDGE, ATTITUDE, AND PERCEPTION OF ANTICIPATING 2022 GLOBAL HUMAN MONKEYPOX INFECTION AMONG INTERNAL MEDICINE RESIDENTS AT PALEMBANG INDONESIA: AN ONLINE SURVEY. *J. Kedokt. Dan Kesehat. Publ. Ilm. Fak. Kedokt. Univ. Sriwij.* **2022**, *9*, 253–262, doi:10.32539/JKK.V9I3.18799.

23. Riccò, M.; Ferraro, P.; Camisa, V.; Satta, E.; Zaniboni, A.; Ranzieri, S.; Baldassarre, A.; Zaffina, S.; Marchesi, F. When a Neglected Tropical Disease Goes Global: Knowledge, Attitudes and Practices of Italian Physicians towards Monkeypox, Preliminary Results. *Trop. Med. Infect. Dis.* **2022**, *7*, 135, doi:10.3390/tropicalmed7070135.

24. Meo, S.A.; Al-Khlaiwi, T.; Aljofan, Z.F.; Alanazi, A.I.; Meo, A.S. Public Perceptions of the Emerging Human Monkeypox Disease and Vaccination in Riyadh, Saudi Arabia: A Cross-Sectional Study. *Vaccines* **2022**, *10*, 1534, doi:10.3390/vaccines10091534.

25. Temsah, M.-H.; Aljamaan, F.; Alenezi, S.; Alhasan, K.; Saddik, B.; Al-Barag, A.; Alhaboob, A.; Bahabri, N.; Alshahrani, F.; Alrabiaah, A.; et al. Monkeypox Caused Less Worry than COVID-19 among the General Population during the First Month of the WHO Monkeypox Alert: Experience from Saudi Arabia. *Travel Med. Infect. Dis.* **2022**, *49*, 102426, doi:10.1016/j.tmaid.2022.102426.

26. Kumar, N.; Ahmed, F.; Raza, M.S.; Rajpoot, P.L.; Rehman, W.; Khatri, S.A.; Mohammed, M.; Muhammad, S.; Ahmad, R. Monkeypox Cross-Sectional Survey of Knowledge, Attitudes, Practices, and Willingness to Vaccinate among University Students in Pakistan. *Vaccines* **2022**, *11*, 97, doi:10.3390/vaccines11010097.

27. Lin, G.S.S.; Tan, W.W.; Chan, D.Z.K.; Ooi, K.S.; Hashim, H. Monkeypox Awareness, Knowledge, and Attitude among Undergraduate Preclinical and Clinical Students at a Malaysian Dental School: An Emerging Outbreak during the COVID-19 Era. *Asian Pac. J. Trop. Med.* **2022**, *15*, 461, doi:10.4103/1995-7645.359787.

28. Winters, M.; Malik, A.A.; Omer, S.B. Attitudes towards Monkeypox Vaccination and Predictors of Vaccination Intentions among the US General Public. *PloS One* **2022**, *17*, e0278622, doi:10.1371/journal.pone.0278622.

29. Harapan, H.; Setiawan, A.M.; Yufika, A.; Anwar, S.; Wahyuni, S.; Asrizal, F.W.; Sufri, M.R.; Putra, R.P.; Wijayanti, N.P.; Salwiyadi, S.; et al. Physicians’ Willingness to Be Vaccinated with a Smallpox Vaccine to Prevent Monkeypox Viral Infection: A Cross-Sectional Study in Indonesia. *Clin. Epidemiol. Glob. Health* **2020**, *8*, 1259–1263, doi:10.1016/j.cegh.2020.04.024.
